# Urinary N-acetyl-beta -D-glucosaminidase and its isoenzymes A & B in workers exposed to cadmium at cadmium plating

**DOI:** 10.1186/1745-6673-2-5

**Published:** 2007-07-20

**Authors:** Ravi Babu Kalahasthi, HR Rajmohan, BK Rajan, Karuna Kumar M

**Affiliations:** 1 Regional Occupational Health Centre (Southern), Indian Council of Medical Research, Bangalore Medical College Campus, Bangalore-560 002, India; 2 Department of studies in Biochemistry, University of Mysore, Mysore, India

## Abstract

**Objective:**

The present study was carried out to determine the effect of cadmium exposure on Urinary N-acetyl-beta -D-glucosaminidase and its isoenzymes A and B in workers exposed at cadmium plating.

**Methods:**

50 subjects using cadmium during cadmium plating formed the study group. An equal number of age-sex matched subjects working in administrative section formed the control group. Urinary cadmium levels were determined by using a flameless atomic absorption spectrophotometer. Urinary N-acetyl-beta -D-glucosaminidase and its isoenzymes A and B were determined by using spectrophotmetric method.

**Results:**

A significant increase of urinary total N-acetyl-beta -D-glucosaminidase and its isoenzymes A and B profiles were noted in study as compared to controls. The levels of urinary N-acetyl-beta -D-glucosaminidase and its isoenzymes A and B profiles were positively and significantly correlated with cadmium levels in urine. Multiple regression analysis was used to assess the effect of urinary cadmium or life style confounding factors (age, BMI, smoking and alcohol consumption) on urinary N-acetyl-beta -D-glucosaminidase and its isoenzymes A and B. The analysis showed that the study subjects who had urine cadmium levels greater than 5 μg/g of creatinine, work duration >15 years, smoking and body mass index variables were significantly associated with urinary total N-acetyl-beta -D-glucosaminidase but not on isoenzymes A&B.

**Conclusion:**

The results presented in this study shows that the increased levels of urinary N-acetyl-beta -D-glucosaminidase observed in cadmium-exposed workers could be used as biomarkers for suggesting preventive measure.

## Background

Cadmium (Cd) is a highly corrosion-resistant metal used extensively for electroplating in general industrial hardware as well as in automotive, electronics, marine and aerospace industries. Cd plating is the process of oxidation of metal articles by the use of Cd-containing acids or bases. The process of Cd plating involves three steps: cleaning, plating and post-treatment of articles. Cd is used as a cadmium oxide in the electroplating of various articles used in the telephone-manufacturing process. The general population is exposed to Cd by food ingestion [1] and smoking [2]. The workers engaged in this process are exposed to Cd by inhalation, ingestion, and dermal contact. Inhalation is the primary route of occupational exposure to metals [3]. Once cadmium enters into human body via inhalation, it is transported to liver and induces the synthesis of metallothionein, a low molecular weight protein. Cadmium bounds to this protein in liver, releases back to the blood and transported to the kidney. In kidney, the cadmium-metallothionein complex passes through the glomeruli and reabsorbed by the proximal tubules. This complex can be broken down by lysosomes and releases unbound cadmium which can again induces the renal synthesis of metallothionein. In workers with short-term exposures to low levels of cadmium, the cadmium bound metallothionein in the kidney provides a protective effect from cadmium toxicity. However, in prolonged exposure the binding process becomes saturated in kidney and leads to increase in unbound cadmium that causes the toxic effects. Studies related to occupational exposure to cadmium at cadmium plating process shown the nasal toxicity and renal tubular dysfunction by using urinary β_2_-microglobulin [4-6]. The urinary β_2_-microglobulin is unstable in acidic urine. The levels of urinary N-acetyl-beta -D-glucosaminidase and its isoenzymes A and B determined in smokers [7], workers exposed to Pb from PVC stabilizers [8]. At present no reports are available regarding occupational exposure to Cd at cadmium plating and its effect on urinary N-acetyl-beta -D-glucosaminidase and its isoenzymes A and B. Therefore, the present study was undertaken to investigate the effect of Cd exposure on urinary N-acetyl-beta -D-glucosaminidase and its isoenzymes A & B in workers involved at cadmium plating.

N-acetyl-beta -D-glucosaminidase is high molecular weight lysosomal enzyme and cannot pass through glomerular ultrafilterate. This enzyme shows high activity in renal proximal tubular cells. The increased level of urine-NAG reflects the proximal tubular dysfunction of the kidney [9]. There are two main isoenzymes (N-acetyl-beta -D-glucosaminidase) found in human kidney [10]. Isoenzyme-A is part of intralysosomal compartment excreted in urine due to exocytosis. Isoenzyme-B is associated to the lysosomal membrane and excreted in urine during tubular damage [11]. These two enzymes are differing in their heat sensitivity. Isoenzyme-A is heat labile whereas isoenzyme-B is heat stable [12]. The separations of the heat stable NAG-B and heat labile NAG-A isoenzymes carried out by heating the urine sample for 30 minutes at 55°C [13]. Tassi et al [14] have separated the N-acetyl-beta -D-glucosaminidase and its isoenzymes (A&B) in Cd-exposed and non-exposed subjects by using DEAE-cellulose chromatography. The present study have determined N-acetyl-beta -D-glucosaminidase and its isoenzymes A &B in Cd-exposed workers and controls by using their heat sensitivity and spectrophotometric method.

## Methods

The study was carried out in 100 male subjects working in a telephone manufacturing plant located in Bangalore (India). These subjects were divided into two groups. The first group formed the study group and consisted of 50 workers engaged in Cd plating with an exposure period ranging from 10 to 18 years. The control group of equal size (50 subjects) was selected from administrative employees of the plant working faraway from the place of work of the study group. A higher level of air borne cadmium concentration was noticed in study area (1.6 μg/m^3 ^in resipable particulate matter) as compared to control area (0.12 μg/m^3^). Subjects of both the groups are matched regarding age and socio-economic status. A standardized questionnaire was used to collect demographic information, work history and habits of all subjects. Subjects with a history of diabetes or hypertension were excluded from the study. Ethical committee has approved the study. Informed consent was obtained from the subjects included in the study.

### Body mass index

Body mass index (BMI) is a measure of body fat based on height and weight of adult men and women. The BMI was calculated by using the formula: weight (kg)/[height (m)]^2 ^with the guidelines of Department of Health and Human Services of National Institute of Health. The body mass index of individuals was expressed in Kg/m^2.^

### Urine cadmium

Urine samples were collected (at the end of the shift) in a metal-free polyethylene bottles. The end shift urine samples were collected form the study and control subjects as per the guidelines of clinical chemistry division of International Union of Pure and Applied Chemistry [15]. They were diluted with equal volume of 0.3 mol/L HNO_3 _and stored at 4°C till the analysis. The Cd level in urine samples was measured by the method of Vesterberg and Wrangskogh [16] using flameless atomic absorption spectrophotometer equipped with graphite furnace (GF-3000) and auto sampler (PAL-3000). The Cd standard curve was linear up to 25 μg/L and detection limit was 0.33 μg/L. The internal standard of Cd was added to urine and analyzed, and a recovery rate of 98.2% was found.

### Total N-acetyl-â-D-glucosaminidase and its Isoenzmes A and B

The levels of urinary N-acetyl-beta -D-glucosaminidase and its isoenzymes A and B were determined by the method of Noto et al [17]. In this method, N-acetyl-beta -D-glucosaminidase reacts with sodium m-cresolsulfonphthaleinyl-N-acetyl-β-D-glucosaminide with release of m-cresolsulfonphthalein (purple Color) and N-acetyl-β-D-glucosaminide. The intensity of color was measured at 580 nm by using a UV-visible spectrophotometer (Shicmadaz Japan model-UV-1601P).

The separation of isoenzymes-A and B was carried out by the method of Chia et al [18]. In this method, urine sample was heated for 30 minutes at 55°C and carried the separation of the heat stable (B) and heat labile (A). The amount of heat labile (A) was calculated by subtracting the heat stable (B) from the total NAG activity. The levels of urinary total N-acetyl-beta -D-glucosaminidase and its isoenzmes A and B were expressed as units per gram of creatinine. One unit of enzyme activity is defined as the amount of enzyme required to catalyze the formation of 1 μmol of m-cresolsulfonphthalein per minute in one liter of sample at 37°C.

The urinary Cd and urinary total N-acetyl-beta -D-glucosaminidase and its isoenzymes A & B were standardized with urinary creatinine concentration measured by Jaffe reaction method of Husdan and Rapoport [18].

### Statistical analysis

SPSS package, version 7.5 for Windows was used for statistical analysis of the data. The student t-test was used to compare the means for age, body mass index, urinary Cd concentration and urine total-NAG and its isoenzymes A&B between the Cd-exposed workers and control group subjects. The χ^2^-test was used to compare the frequency distribution of Cd-exposed workers and control group subjects. Pearson's correlation coefficient was used to find out the association between urinary Cd levels and urinary NAG and its isoenzymes A&B. ANOVA was used to compare urinary NAG and its isoenzymes A&B with variables. Stepwise multiple regression analysis was used to assess the effect of variables on urinary NAG and its isoenzymes A&B parameters.

## Results

Table-1 shows the demographic details of study and control groups. The average age, body mass index and duration of work of study and control groups were suitably matched. The frequency distributions of life style confounding factors showed no significant differences between the two groups.

**Table 1:** Demographic details of cadmium-exposed and controls

Variables	Cadmium exposed (N = 50)	Control group (N = 50)
Age (Years)	42.9 ± 2.38^a^	41.8 ± 3.45
Work duration (years)	13.5 ± 2.73	14.2 ± 1.82
Body mass index (Kg/m^2^)	26.4 ± 2.83	26.3 ± 2.95
Smoking		
No	43(86)^b^	44(88)
Yes	7(14)	6(12)
Alcohol consumption		
Usually	1(2)	6(12)
Sometimes	5(10)	7(14)
Never	44(88)	37(74)

^a ^Mean ± standard deviation

^b ^Number of persons

Figures in parenthesis indicates percentages of subjects

The average levels of urinary Cd and uurinary N-acetyl-beta -D-glucosaminidase and its isoenzymes-A and B in study and control group subjects are presented in Table-2. The levels of urinary Cd and urinary total-N-acetyl-beta -D-glucosaminidase and isoenzymes-A and B were significantly higher in study subjects when compared to controls.

**Table 2:** Urine cadmium, total NAG and isoenzymes A and B in cadmium exposed and controls.

Variables	Cadmium exposed (N = 50)	Control group (n = 50)
Urine cadmium (μg/g of creatinine)	7.04 ± 3.49***	3.93 ± 0.70
Urinary Total NAG (U/g of creatinine)	5.09 ± 2.00***	2.77 ± 0.66
Urinary NAG-A (U/g of creatinine)	3.65 ± 1.55***	1.86 ± 0.68
Urinary NAG-B (U/g of creatinine)	1.44 ± 0.67***	0.90 ± 0.30

Values are mean ± standard deviation

***P < 0.001

Table-3 showed the effects of smoking on urinary cadmium excretion in Cd-exposed workers and controls. The comparison of cadmium-exposed smoker with cadmium-exposed non-smokers and Cd-non exposed-smokers were made. A significant (P = 0.020) difference was noticed between cadmium exposed-smokers and Cd-non-exposed smokers of control. The comparison between Cd-exposed non-smokers and Cd-non-exposed-non-smokers showed significant (P = 0.030) differences. The synergetic effect of smoking on urinary cadmium excretion showed in Cd-exposed-smokers as compared with Cd-non-exposed smokers.

**Table 3:** the effect of smoking on urinary cadmium excretion in Cd-exposed workers and controls

Category	(n)	Urine cadmium (μg/g of creatinine)
Cd-exposed smokers	(7)	7.3 ± 3.0
Cd-exposed-Non-smokers	(43)	5.7± 4.7
Cd-non-exposed-smokers of Control	(6)	3.9 ± 0.7
Cd-non-exposed-non smokers of controls	(44)	3.1 ± 0.4

Values are mean ± standard deviation

The correlations coefficients (r) between urinary Cd and urinary N-acetyl-beta -D-glucosaminidase and its isoenzymes-A and B in subjects are presented in Table-4. A positive and significant correlation coefficients (r) were observed between urinary Cd levels and urinary total-N-acetyl-beta -D-glucosaminidase and its isoenzymes-A & B. These correlation coefficients (r) were significant at P < 0.01.

**Table 4:** Correlation coefficient (r) between urine cadmium and urinary total N-acetyl-beta -D-glucosaminidase and isoenzymes A and B. (N = 100)

Variables	Urine cadmium	Urinary Total NAG	Urinary NAG-A	Urinary NAG-B
Urine cadmium (μg/g of creatinine)	1.000	-	-	-
Urinary Total NAG (U/g of creatinnine)	0.738**	1.000	-	-
Urinary NAG-A (U/g of creatinnine)	0.710**	0.966**	1.000	-
Urinary NAG-B (U/g of creatinnine)	0.563**	0.751**	0.555**	1.000

** Correlation is significant at P < 0.01

Table-5 shows the results of univariate analysis of variables that affect the urinary total-N-acetyl-beta -D-glucosaminidase and isoenzymes-A and B. The levels of urinary total-N-acetyl-beta -D-glucosaminidase and its isoenzymes-A and B were affected significantly in subjects who had urinary Cd levels greater than 5 μg/g of creatinine. No significant differences were noticed for variables such as age, BMI, consumption of alcohol, smoking and subjects who had urinary Cd level less than 5 μg/g of creatinine.

**Table 5:** Univariate analysis of the variable that affect the urinary total N-acetyl-beta -D-glucosaminidase and its isoenzymes A and B (N = 100).

Variables	(n)	Urinary Total NAG (U/g of creatinnine)	Urinary NAG-A (U/g of creatinnine)	Urinary NAG-B (U/g of creatinnine)
Age (years)				
≥45	(92)	3.67 ± 1.68	2.65 ± 1.48	1.02 ± 0.48
>45	(8)	3.95 ± 1.90	2.77 ± 1.50	1.18 ± 0.59
Work duration (years)				
10–15	(62)	3.88 ± 1.73	2.66 ± 1.30	1.10 ± 0.57
>15	(39)	4.02 ± 2.13	2.92 ± 1.79	1.18 ± 0.56
BMI (Kg/m^2^)				
18.5–24.9	(32)	3.83 ± 1.87	2.63 ± 1.50	1.20 ± 0.60
25.0–29.9	(54)	4.03 ± 2.03	2.85 ± 1.60	1.18 ± 0.62
>30	(14)	3.77 ± 1.32	2.70 ± 1.04	1.07 ± 0.40
Smoking				
Yes	(13)	4.12 ± 2.27	3.02 ± 1.83	1.10 ± 0.54
No	(87)	3.90 ± 1.83	2.72 ± 1.50	1.18 ± 0.59
Alcohol Consumption				
Usually	(7)	3.32 ± 0.78	2.37 ± 0.68	0.95 ± 0.40
Sometimes	(12)	3.02 ± 1.27	1.98 ± 0.92	1.04 ± 0.50
Never	(81)	4.12 ± 1.98	2.91 ± 157	1.21 ± 0.60
Urine cadmium				
≤5	(67)	3.00 ± 1.07	2.05 ± 0.92	0.95 ± 0.40
>5	(33)	5.83 ± 1.74***	4.20 ± 1.40***	1.63 ± 0.63***

***P < 0.001

Table-6 shows the results of stepwise multiple regression analysis of variables that affect urinary total-N-acetyl-beta -D-glucosaminidase and its isoenzymes-A and B. The variables included in the regression model were age (1 = ≤45 years and 2 = >45 years), The work duration (years) of subjects were categorized into two groups based on duration of work (1 = 10–15 years of exposure) and (2 = >15 years of exposure), body mass index (1 = 18.5–24.9 kg/m^2^, 2 = 25–29.9 kg/m^2 ^and 3 = ≥30 kg/m^2^), Alcohol consumption (1 = Usually, 2 = sometimes and 3 = never). The level of urinary Cd was categorized into two groups (1 = ≤5 μg/g of creatinine and 2 = >5 μg/g of creatinine) as per the recommendation of international standards: WHO-1999[20] and ACGIH-2006[21]. Multiple regression analysis showed that the age >45 years had a significant influence (57%) on urinary total-N-acetyl-beta -D-glucosaminidase activity but not on isoenzymes-A & B. The subjects who had work duration 10–15 years influenced 34% on urinary total-N-acetyl-beta -D-glucosaminidase. In subjects who had work duration >15 years showed 82% association on urinary total-N-acetyl-beta -D-glucosaminidase. Both categories of work duration did not showed any significant association on isoenzymes-A and B. Subjects with body mass index of 18.5–24.9 kg/m^2 ^(40%) 25–29.9 kg/m^2 ^(63%) and ≥30 kg/m^2 ^(60%) showed a significant association with excretion of urinary total-N-acetyl-beta -D-glucosaminidase activity. Smokers had significant influence (53%) on urinary total-N-acetyl-beta -D-glucosaminidase activity. Subjects who had urinary Cd levels greater than 5 μg/g of creatinine appeared to have an influence (52%) on urinary total-N-acetyl-beta -D-glucosaminidase activity. The variables such as age, BMI, smoking status, alcohol consumption and urine cadmium did not show any significant influence on isoenzymes-A and B

**Table 6:** Multiple regression analysis of variables that affect the total N-acetyl-beta -D-glucosaminidase and its isoenzymes A and B (N = 100).

Variables	Urinary Total NAG (U/g of creatinine) β (P-value)	Urinary NAG-A (U/g of creatinine) β (P-value)	Urinary NAG-B (U/g of creatinine) β (P-value)	R^2^
Age (years)				
>45	1.173(0.000)*	-0.197(0.471)	0.078(0.471)	57
Work duration (years)				
10–15	0.860(0.000)*	-0.446(0.271)	0.205(0.271)	34
>15	1.463(0.000)*	-0.297(0.327)	0.096(0.327)	82
BMI (Kg/m^2^)				
18.5–24.9	0.955(0.000)*	-0.221(0.669)	0.089(0.669)	40
25.0–29.9	1.226(0.000)*	0.055(0.875)	-0.021(0.875)	63
>30	1.571(0.001)*	-0.079(0.797)	0.031(0.797)	60
Smoking (Cigarettes/day)				
Yes	1.087(0.000)*	-0.138(0.614)	0.057(0.614)	53
Alcohol Consumption (Drinks/week)				
Never	1.185(0.000)*	0.170(0.064)	0.170(0.064)	57
Urine cadmium (μg/g creatinine)				
>5	0.967(0.000)*	0.064(0.870)	-0.029(0.870)	52

β(P-values) = regression coefficient (P-value of regression coefficient).

a = Units per gram of creatinine

b = regression coefficient and p-value* indicated in brackets significant at P < 0.05

c = regression coefficient and p-value indicated in brackets without mark not significant

## Discussion

The present study assessed the effect of Cd exposure on urinary total-N-acetyl-beta -D-glucosaminidase and its isoenzymes A and B in workers involved at cadmium plating. Since the urinary Cd levels were associated with cadmium contents in kidney [22,23], the present study used the urinary Cd levels as indicator of body burden. The absorption of cadmium was quantified in the urine samples of Cd-exposed workers and control group. During the present study it was noted that the urinary Cd levels in Cd-exposed workers showed significantly higher when compared to the controls. Yassin and Martonik [24] have reported urinary Cd levels ranging from 0.01 to 15.57 μg/L in the US working population. It is comparable with the present results (0.5 – 17 μg Cd/g creatinine).

There are two main isoenzymes (N-acetyl-beta -D-glucosaminidase) found in human kidney. Isoenzyme-A is part of intralysosomal compartment excreted in urine due to exocytosis. Isoenzyme-B is linked to the lysosomal membrane and excreted in the urine during tubular damage. The present study assessed the urinary total-NAG and its isoenzymes-A & B in workers exposed to cadmium at cadmium plating process in order to find the status of exocytosis and tubular damage of kidney.

During the present study it was noted that the urinary total-N-acetyl-beta -D-glucosaminidase and its isoenzymes-A & B levels were significantly higher in Cd-exposed workers when compared to controls. The levels of urinary total-N-acetyl-beta -D-glucosaminidase and its isoenzymes-A and B were positively and significantly correlated with urinary cadmium levels. Since, the excretion of uurinary N-acetyl-beta -D-glucosaminidase and its isoenzymes-A & B are related to life style confounding factors (age, body mass index, smoking status and alcohol consumption). The present study assessed the association between urinary total-N-acetyl-beta -D-glucosaminidase and its isoenzymes-A and B with life style confounding factors.

Efskind et al [25] reported an association between urinary N-acetyl-beta -D-glucosaminidase and age. Stengel et al [26] reported that the subject's age, body mass index, and smoking had significantly influence on urinary total-N-acetyl-beta -D-glucosaminidase. The present study also reported similar association but not on isoenzymes-A and B.

Tassi et al [14] reported higher levels of isoenzyme-B in cd-exposed workers with urinary cd levels ranging from 2 μg/g creatinine to ≤10 μg/g creatinine. Using DEAE-cellulose chromatography separated urinary N-acetyl-beta -D-glucosaminidase isoenzymes. Jin et al [27] reported dose-dependent increase of NAG and NAG B contents in urine related to urinary Cd and calculated Cd-uptake. Bernard et al [28] reported the association between NAG-B and urinary cadmium showed no evidence of a threshold. During the present study it was noted that the subjects who had urine Cd levels greater than 5 μg/g of creatinine had influenced only on urinary total-N-acetyl-beta -D-glucosaminidase but not isoenzymes A and B. These findings were appropriate with workshop of biomarkers of nephrotoxicity [9].

## Conclusion

The urinary N-acetyl-beta -D-glucosaminidase and its isoenzymes-A and B levels were significantly higher in Cd-exposed workers when compared to controls. The levels of urinary total-N-acetyl-beta -D-glucosaminidase and its isoenzymes-A and B were positively and significantly correlated with urinary Cd levels. However in multiple regression analysis showed that the subjects who had urinary Cd levels greater than 5 μg/g of creatinine significantly influenced only the urinary total-N-acetyl-beta -D-glucosaminidase but not on isoenzymes A and B. Hence, urinary total-N-acetyl-beta -D-glucosaminidase activity could be used as biomarker for renal tubular dysfunction in Cd-exposed workers.

## References

[B1] Puklova  V, Batariova A, Cerna M, Kotlik B, Kratzer K, Melichercik J, Ruprich J, Rehurkova I, Spevakova V (2005). Cadmium exposure pathways in the Czech urban population. Cent Eur J Public Health.

[B2] Sisman AR, Bulbul M, Coker C, Onvural B (2003). Cadmium exposure in tobacco workers: possible renal effects. J trace Elem Med Biol.

[B3] Kelleher P, Pacheco K, Newman LS (2000). Inorganic dust pneumonias: The metal related parenchyma disorders. Environ Health Perspect.

[B4] Rydzewski B, Sulkowski W, Miarzynska M (1998). Olfactory disorders induced by cadmium exposure: a clinical study. Int J Occup Med Environ Health.

[B5] Sunderman FW (2001). Nasal toxicity, carcinogencity and olfactory uptake of metals. Ann Clin Lab Sci.

[B6] Zwennis WC, Fransen AC (1992). Assessment of occupational exposure to cadmium in the Netherlands, 1980–1989. Am J Ind Med.

[B7] Roszczenko A, Galazyn-Sidorczuk M, Brzoska MM, Moniuszko-Jakoniuk J, Zwierz K (2004). Select parameters of renal function in smokers in correlation with the exposure to cadmium. Przegl Lek.

[B8] Lim YC, Chia KS, Ong HY, Nag V, Chew YL (2001). Renal dysfunction in workers exposed to inorganic lead. Ann Acad Med.

[B9] Mueller PW, Robert, Price G, William, Finn F (1998). New approaches for detecting thresholds of human nephrotoxicity using cadmium as an example. Environ Health Perspectives.

[B10] Dance N, Price RG, Cattel WR (1970). The excretion of N-acetyl-D-glucosaminidase and B-galactosidase by patients with renal disease. Clin Chim Acta.

[B11] Gibey R, Dupond JL, Henry JC (1984). Urinary N-acetyl-D-glucosaminidase (NAG) isoenzymes profiles: a toll for evaluating nephrptoxicity of amino glycosides and cephalosporins. Clin Chim Acta.

[B12] Dance N, Price RG, Robinson D, Stirling JL (1969). B-galactosidase, B-glucosidase and N-acetyl-D-glucosamnidase in human kidney. Clin Chim Acta.

[B13] Tan C, Cheng LY, Chia KS, Jeyaratnam J (1991). Separation of N-acetyl-beta-D-glucosaminidase isoenzymes profile by heat treatment: its reproducibility. Journal of Medical Laboratory Science.

[B14] Tassi C, Abbritt G, Mancuso F, Morucci P, Feligioni L, Muzi G (2000). Activity and isoenzymes profile of N-acetyl-beta-D-glucosaminidase in urine from workers exposed to cadmium. Clin Chim Acta.

[B15] International union of pure and applied chemistry (IUPAC), clinical chemistry division (1995). Sample collection guidelines for trace elements in blood and urine. Pure App Chem.

[B16] Vesteberg O, Wrangskogh K (1978). Determination of urine by graphite-furnace atomic absorption spectroscopy.

[B17] Noto A, Ogawa Y, Moni S (1983). Simple and rapid spectrophotometry of urinary N-acetyl-D-glucosaminidase with use of a new chromogenic substrate. Clin Chem.

[B18] Chia KS, Mutti A, Tan C, Ong HY, Jeyaratnam J, Ong CN, Lee E (1994). Urinary N-acetyl-beta-D-glucosaminidase activity in workers exposed to inorganic lead. Occup Environ Med.

[B19] Husdan H, Rapoport A (1969). Estimation of creatinine in Jaffe reaction. Clin Chem.

[B20] WHO (1999). Cadmium. Recommended Health-Based Limits in Occupational Exposure to Heavy Metals.

[B21] American Conference of Governmental Industrial Hygienists (2006). The documentation of Threshold Limit Values and Biological Exposure Indices of chemical substances and physical agents. Cincinnati. USA.

[B22] Orlowski C, Piotrowski JK, Subdys JK, gross A (1998). Urinary cadmium as indicator of renal cadmium in humans: an autopsy study. Human & Experimental toxicology.

[B23] Borjesson J, Gerhardsson L, Sxhetz A, Perfekt R, Maatsson S, Skerfving S (2001). Kidney cadmium as compared to other markers of cadmium exposure in workers at s secondary smelter. Am J Ind Med.

[B24] Yassin AS, Martonik JF (2004). Urinary cadmium levels in the US working population. 1984–1994. J Occup Environ Hyg.

[B25] Efskind J, Ellingsen DG, Hartman A, Thomassen Y, Ulvik RJ, Gaarder PI, Solberg TB (2006). Renal function of chloralkali workers after the cessation of exposure to mercury vapor. Scand J Work Environ Health.

[B26] Stengel B, Watier L, Chouquet C, Cenee S, Philippon C, Hemon D (1999). Influence of renal biomarker variability on the design and interpretation of occupational or environmental studies. Toxicol Lett.

[B27] Jin T, Nordberg G, Wu X, Ye T, Kong Q, Wang Z, Zhuang F, Cai S (1999). Urinary N-acetyl-beta-D-glucosaminidase isoenzymes as biomarker of renal dysfunction caused by cadmium in a general population. Environ Res.

[B28] Bernard A, Thielemans N, Roels H, Lauwerys R (1995). Association between NAG-B and cadmium in urine with no evidence of a threshold. Occup Environ Med.

